# Epidemiological Trends and Hotspots of Other Infectious Diarrhea *(OID)* in Mainland China: A Population-Based Surveillance Study From 2004 to 2017

**DOI:** 10.3389/fpubh.2021.679853

**Published:** 2021-07-22

**Authors:** Can Chen, Zhou Guan, Chenyang Huang, Daixi Jiang, Xiaoxiao Liu, Yuqing Zhou, Danying Yan, Xiaobao Zhang, Yiyi Zhou, Cheng Ding, Lei Lan, Yushi Lin, Jie Wu, Lanjuan Li, Shigui Yang

**Affiliations:** State Key Laboratory for Diagnosis and Treatment of Infectious Diseases, National Clinical Research Center for Infectious Diseases, Collaborative Innovation Center for Diagnosis and Treatment of Infectious Diseases, The First Affiliated Hospital, Zhejiang University School of Medicine, Hangzhou, China

**Keywords:** other infectious diarrhea, epidemiological trends, hotspots, joinpoint regression, space-time analyses

## Abstract

**Background:** The incidence of other infectious diarrhea *(OID)* ranked second in class C notifiable disease in China. It has posed a great threat to public health of all age groups. The aim of this study was to investigate the epidemiological trends and hotspots of OID in mainland China.

**Materials and Methods:** Incidence and mortality data for OID stratified by date, age and region from 2004 to 2017 was extracted from the data-center of China public health science. Joinpoint regression and space-time analyses were performed to explore the epidemiological trends and hotspots of OID.

**Results:** The average annual incidence of OID was 60.64/100,000 and it showed an increased trend in the mainland China especially after 2006 (APC = 4.12, 95 CI%: 2.06–6.21). Children of 0–4 year age group accounts for 60.00% (5,820,897/11,414,247) of all cases and its incidence continuously increased though 2004–2017 (APC = 6.65, 95 CI%: 4.39–8.96). The first-level spatial and temporal aggregation areas were located in Beijing and Tianjin, with the gathering time from 2005/1/1 to 2011/12/31 (RR = 5.52, LLR = 572893.59, *P* < 0.001). The secondary spatial and temporal aggregation areas covered Guangdong, Guangxi, Hainan and Guizhou from 2011/1/1 to 2017/12/31 (RR = 1.98, LLR = 242292.72, *P* < 0.001). OID of Tianjin and Beijing presented a decreased trend since 2006. However, the incidence of OID in Guangdong, Guangxi, Hainan and Guizhou showed increased trends through 2004–2017.

**Conclusion:** Our study showed that OID showed a constantly increasing trend and brought considerable burden in China especially in the 0–4 age group. The high-risk periods and clusters of regions for OID were identified, which will help government develop disease-specific and location-specific interventive measures.

## Introduction

Infectious diarrhea is one of the most common infectious diseases around the world and acts as an important indicator to regional hygiene, food safety and public health (1). In 2010, the diarrhoeal disease ranked second in the global burden of infectious diseases and in 2015, about 2.3 billion people had experienced diarrhea worldwide (2). It was also a major cause of malnutrition and mortality among children under 5 in developing countries (3). China is one of 15 countries with a high disease burden of pneumonia and diarrhea (4). In China, other infectious diarrhea *(OID)* was defined as infectious diarrhea other than cholera, dysentery, typhoid/paratyphoid fever (5). Due to the wide spectrum of pathogens and lack of effective vaccine protection, many regions presented high incidences (6). In 2017, the incidence of OID ranked second in class C notifiable diseases in China (7). It has posed a great threat to public health of all age groups, especially to infants and young children, which therefore caused a heavy economic burden in China.

Although many researches has pictured the epidemiological features of OID in China, the majority of these studies were based on the region-specific level (8, 9). Therefore, in order to further describe the overall epidemic characteristics and trends of OID, we systematically analyzed the reported cases of OID from 31 provinces in China. Meanwhile, we then performed the space-time analyses to identify the hotspots of OID. The results of this study would provide a theoretical basis for OID prevention and control in China.

## Materials and Methods

### Data Sources

The incidence and mortality data for infectious diarrhea from 2004 to 2017 were obtained from the data-center of China public health science, which is the main data center of the National Population Health Science data sharing platform in China and covered a population of about 1.3 billion people from 31 provinces and regions in mainland China (10).

### Data Collection

The definition of OID cases in our study followed the criteria issued by the Ministry of Health of the People's Republic of China. In each level medical institution, once a OID case identified, clinicians complete a standard case report card for infectious diseases. The field investigations were performed using a standardized form.

To assess the epidemiological trends and hotspots of OID in mainland China, the data of OID including the number of cases and deaths, the incidence and mortality were stratified by date (month and year), age and region.

## Statistical Analysis

### Epidemiological Trends Analysis

We used joinpoint regression model to examine incidence trends of OID from 2004 to 2017. The annual percentage changes *(APCs)* with their 95% confidence interval *(CI)* were obtained for each trend segment (11). We used Z test to assess whether APCs was significant (*P* < 0.05), and the trends were further described as increased or decreased when the APCs were positive or negative, respectively. While, the trends were considered as stable when the APCs values were not significant (*P* ≥ 0.05).

### Seasonal Decomposition Analysis

The seasonal decomposition analysis was based on the seasonal trend decomposition using SEATS (Seasonal Extraction in ARIMA Time Series) (12), which filters the trend and seasonal component from the time series data and decomposes into three components: trend (T), seasonal (S), and remainder or random (R). The equation can be described as follows. Yt = Tt + St + Rt (12). In this study, Yt is the number of OID cases. t is time in the unit of month.

### Spatial Autocorrelation Analysis

Spatial autocorrelation refers to the potential interdependence of some variables between observed data in the same distribution area. The global Moran's I and local Moran's I were used to measure spatial autocorrelation (13). The global Moran's I ranging from −1 to 1 was used to detect the degree of spatial autocorrelation of research object from the whole region (14). The local Moran's I was used to explore the spatial position of clustering. According to the results of local Moran's I, it presented four categories results including high-high cluster, low-low cluster, high-low cluster, and low-high cluster (15). Z test was used to assess the significant difference.

### Spatial and Temporal Aggregation Analysis

The retrospective spatiotemporal scan statistic based on the discrete Poisson model was applied to detect the space-time cluster of OID in China (16). The dynamic space-time two-dimensional cylinder scanning window was constructed to scan geographic units and time within the study area. The null hypothesis presumed that the window area and outside areas have the same relative risk *(RR)* of incidence. The actual and theoretical incidence numbers inside and outside the scanning window were used to calculate the log likelihood ratio *(LLR)*. The cluster was classified according to the LLR value (e.g. secondary cluster 1, 2) (17). Monte Carlo simulation was used to evaluate statistical significance. We used Microsoft Excel 2016 for data extraction, sorting, and cleaning, and R (version 3.2.3), and SatScan (version 9.5), Joinpoint (version 4.8.0.1) for further data analysis.

## Results

### The Incidence and Trend of Other Infectious Diarrhea in Mainland China From 2004 to 2017

**Figure 1:**
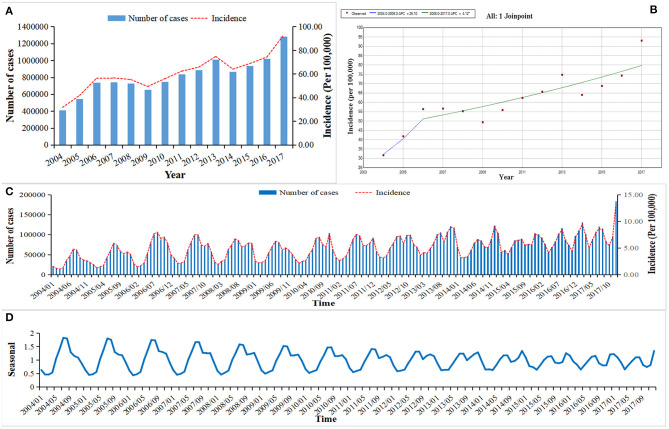
The incidence and trend of other infectious diarrhea in mainland China from 2004 to 2017. **(A)** The annual incidence of OID from 2004 to 2017. **(B)** The Joinpoint analysis of OID from 2004 to 2017. **(C)** The monthly incidence of OID from 2004 to 2017. **(D)** Seasonal decomposition analysis of OID from 2004 to 2017.

A total of 11,414,247 OID cases were reported in mainland China from Jan 1, 2004 to Dec 31, 2017. The average annual incidence was 60.64/100,000, from 2004 (31.70/100,000) to the 2017 (93.10/100,000), (Figure 1A). The incidence of OID presented an increased trend in mainland China especially after 2006 (APC = 4.12, 95CI%: 2.06–6.21, *P* < 0.05), (Figure 1B). Before 2013, two peaks of OID showed up during June to August and September to November, but it turned to the June to August and December to February (Figures 1C,D).

### The Age Group Distribution and Trend of Other Infectious Diarrhea From 2004 to 2017

**Figure 2:**
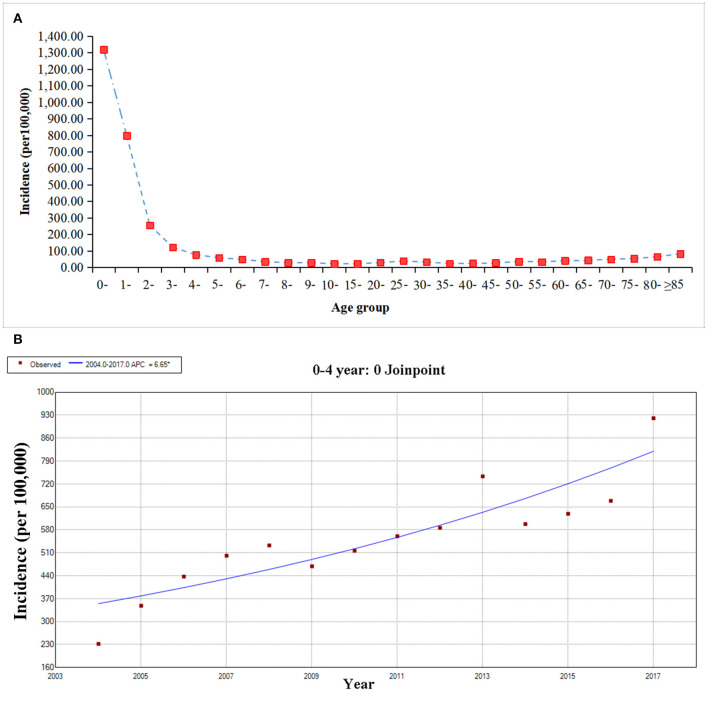
The age group distribution and trend of other infectious diarrhea from 2004 to 2017. **(A)** The age group distribution of OID. **(B)** The Joinpoint analysis of OID in the age of 0–4 year.

The age group of 0- years showed the highest average annual incidence of 1318.10/100,000. Children in 0–4 years group were most at-risk group infected with OID, accounting for 60.00% (5,820,897/11,414,247) of all cases. With the increase of age, the incidences showed a downward trend, and after the four-year age group, the incidences were relatively stable (Figure 2A). The results of Joinpoint analysis showed that the incidence of 0–4 age group continuously increased through 2004–2017 (APC = 6.65, 95CI%: 4.39–8.96, *P* < 0.05) (Figure 2B).

### Geographical Distribution Characteristics of Other Infectious Diarrhea

**Figure 3:**
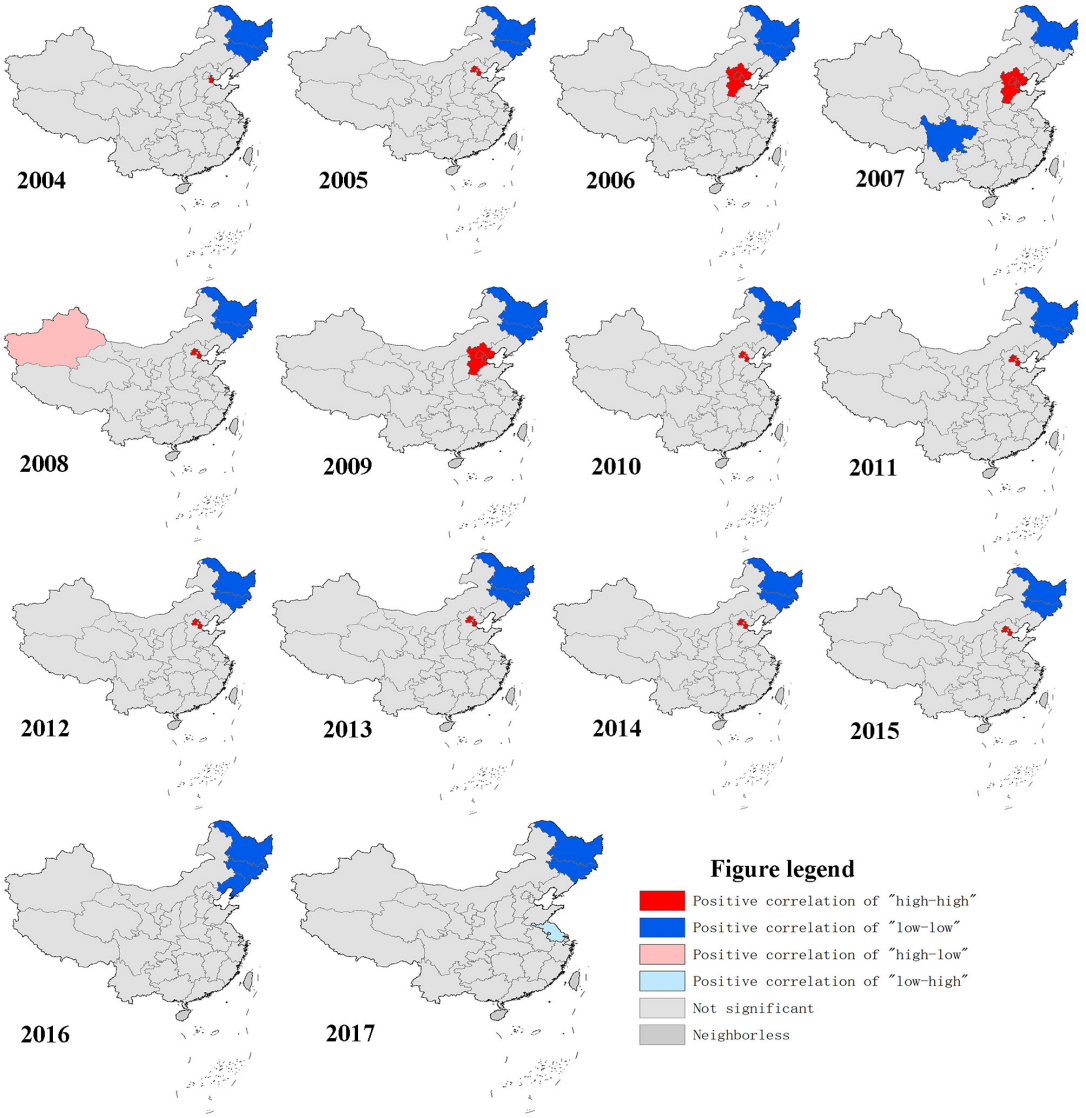
Geographical distribution characteristics of other infectious diarrhea.

**Figure 4:**
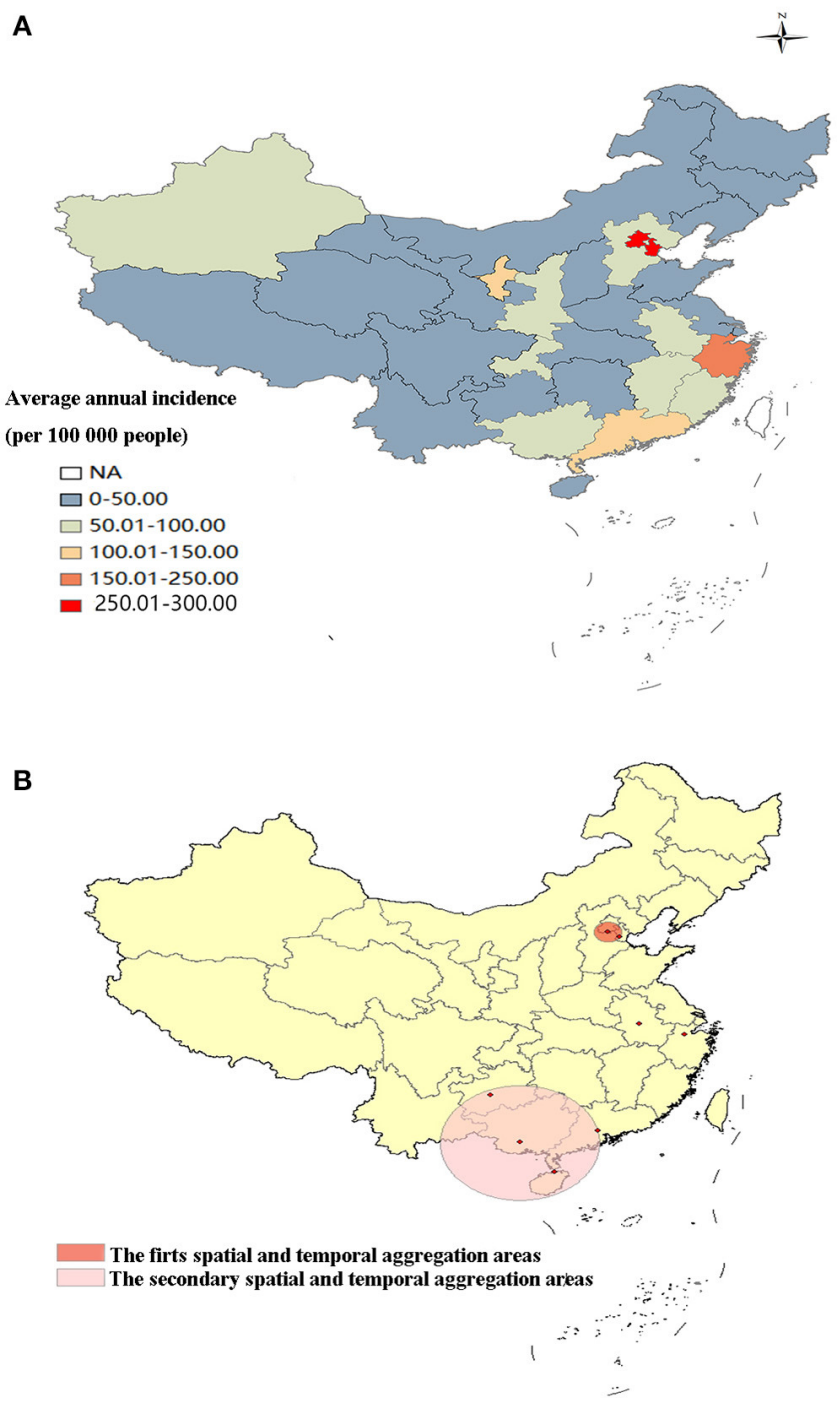
Spatial and temporal aggregation of other infectious diarrhea. **(A)** Geographical distribution of OID in mainland China. **(B)** Spatial and temporal aggregation of OID from 2004 to 2017.

During 2004–2017, The top three incidence regions of OID were Tianjin (289.33/100,000), Beijing (253.67/100,000) and Zhejiang province (200.34/100,000). The OID was higher in the eastern China (83.65/100,000) than central (43.79/100,000) and western China (47.95/100,000), (Figure 4A, Supplementary Table 1). The global spatial autocorrelation analysis results demonstrated a positive correlation during the 2004-2017 (Supplementary Table 2). Then, local spatial autocorrelation at the provincial and autonomous levels were further analyzed. The high-high aggregation areas of OID were found in Tianjin and Beijing from 2004–2015. The low-low aggregation areas were found in Heilongjiang and Jilin from 2004 to 2017 (Figure 3).

### Spatial and Temporal Aggregation Analysis of Other Infectious Diarrhea

Two spatial and temporal aggregation areas were revealed according to spatial and temporal aggregation analyses. The first-level spatial and temporal aggregation areas were distributed in Beijing and Tianjin, with the gathering time in 2005/1/1 to 2011/12/31. The actual number of cases reported in the regions was 658,479, which was much higher than that of the number of expected cases, that is, 125,134 (RR = 5.52, LLR = 572893.59, *P* < 0.001). The secondary spatial and temporal aggregation areas covered four provinces from 2011/1/1 to 2017/12/31. The areas included Guangdong, Guangxi, Hainan and Guizhou. The actual number of cases reported in the region was 1,396,809, but the number of expected cases was 749,523 (RR = 1.98, LLR = 242,292.72, *P* < 0.001), (Figure 4B).

**Figure 5:**
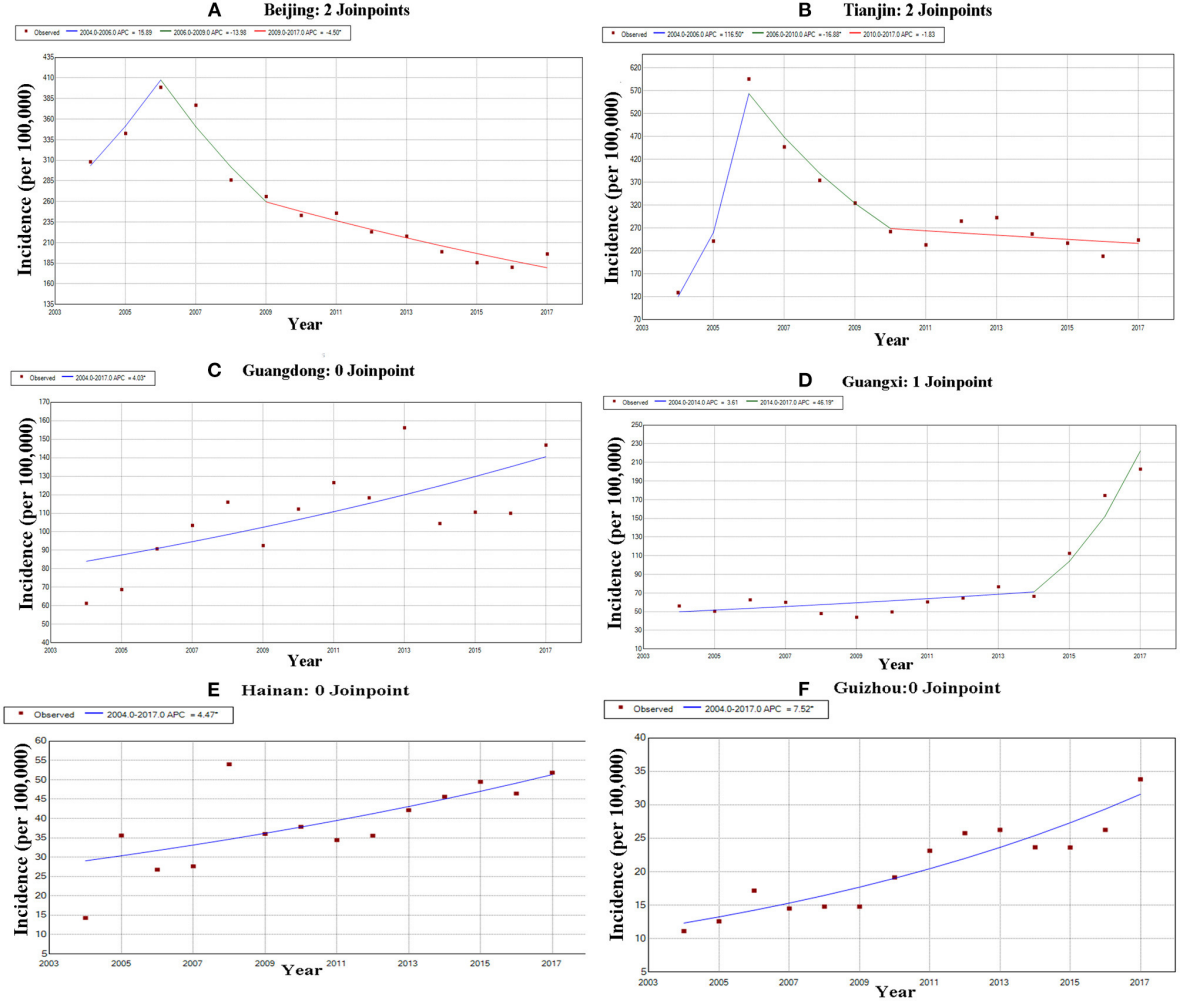
The trends of other infectious diarrhea in hotspots. **(A)** The Joinpoint analysis of OID in Beijing from 2004 to 2017. **(B)** The Joinpoint analysis of OID in Tianjin from 2004 to 2017. **(C)** The Joinpoint analysis of OID in Guangdong from 2004 to 2017. **(D)** The Joinpoint analysis of OID in Guangxi from 2004 to 2017. **(E)** The Joinpoint analysis of OID in Hainan from 2004 to 2017. **(F)** The Joinpoint analysis of OID in Guizhou from 2004 to 2017.

The results of Joinpoint analysis by areas showed that OID showed an increasing trend in most provinces (21/31) (Supplementary Table 1). In Tianjin and Beijing, the OID presented a decreasing trend since 2006. However, the incidence of OID in Guangdong, Guangxi, Hainan and Guizhou showed an increased trend throughout 2004–2017 (Figure 5).

## Discussion

In this study, we investigated the epidemic trend and distribution characteristics of 11,414,247 OID cases in mainland China during a 14-year time period. Our results demonstrated that OID presented a constantly increased trend in mainland China and in 0–4 age group from 2004 to 2017. Two levels hotspots were found. Our findings will provide scientific evidence to policy maker to better prevent and control OID in the following stage.

The expanding of pathogenic spectrum, especially viral pathogens, has promoted the prevalence of OID and caused increasing outbreaks and public health emergencies which usually brought a large number of cases (18). In recent years, as the development of infectious disease surveillance and reporting system, the OID cases were more likely to be detected and reported. The prevalence of OID shifted from summer and autumn peaks to summer and winter peaks since 2013. This might attribute to the increased incidence of viral infectious diarrhea such as rotavirus and norovirus which clustered in winter (19). In 2005–2019, the four most common pathogens of OID in China were rotavirus (85.74%), adenovirus (4.28%), salmonella (3.58%) and norovirus (2.82%) (20). Since 2013, norovirus was reported as the dominant pathogeny in OID outbreaks in China, and rotavirus also increased rapidly in children under 5 years of age (21, 22). The OID incidence of 0–4 years age group showed the increased trends in China. On the one hand, once children are infected, the symptoms are relatively severe due to immature immune system (23). Additionally, parents are more likely to seek medical treatments for their children and then the cases were more likely to be reported. On the other hand, as proportion of viral pathogens in OID increased, children under 5 years old were more vulnerable (24).

In previous studies, diarrhea was found presenting a specific temporal and spatial distribution which usually clustered spatially in different geographical locations (25–27). Several factors including sociodemographic variables, personal hygiene, and environmental and climatic changing were considered to be associated with the incidence of diarrhea (28, 29). Spatiotemporal aggregation of OID in China could be divided into two stages. The first hotspots were detected in the Beijing-Tianjin through 2005–2011. The two sites are both developed areas in China. In the early phase of development and urbanization, it attracted larger floating population characterized by low immune systems, poor living environments and living conditions, and poor health and knowledge of epidemic prevention measures which might cause OID to spread easily (30, 31). As the improvement of the environment and the management of mobile population, risks of infection by various pathogens would reduce. In the Joinpoint regression, the incidence of OID in Beijing-Tianjin decreased since 2006. The second hotspots was identified in Guangdong, Guangxi, Hainan and Guizhou through 2011–2017. The OID in those region showed an increased trend through 2004–2017. In weng's study, public health emergencies caused by OID were found mainly clustered in the Guangdong, Guangxi and Fujian provinces (18). South China is closer to the equator with a subtropical monsoon and tropical monsoon climate, which are characterized by high humidity, temperature, rainfall, and wind speed. And this also facilitated OID transmission (31). Furthermore, residents of coastal areas usually have the habit of eating raw seafood, which also contains a wide variety of pathogens such as rotavirus, norovirus, and vibrio parahaemolyticus (32, 33). Our results show that the incidences of OID in China has a clear population, seasonal and regional distribution. The comprehensive prevention and control measures should be implemented to reduce the incidences of OID before the epidemic peaks. The specific hotspots, highly risk groups should be considered as the priority of prevention and control. At the same time, the monitoring of pathogens should be conducted to further clarify the epidemic characteristics of OID in each jurisdiction. For some pathogens such as rotavirus could be prevented by vaccines. The knowledge should be strengthened to improve the coverage of vaccines, especially in children (34).

## Conclusions

Our study demonstrated that the incidence of OID continuously increased in mainland China especially in 0–4 years age group. The seasonal peak of OID prevalence shifted from summer and autumn to summer and winter since 2013. Currently, Guangdong, Guangxi, Hainan and Guizhou were identified as the hotspots of OID in mainland China. The high-risk periods and clusters of regions for the OID were identified which will help governments to develop disease-specific and location-specific intervention measures.

## Data Availability Statement

The original contributions presented in the study are included in the article/Supplementary Material, further inquiries can be directed to the corresponding author/s.

## Author Contributions

SY, LJL, and JW: designed the study. CC, ZG, CH, DJ, XL, YZ, DY, XZ, YZ, and YL: collected data. CC, ZG, and CH: analyzed data and interpreted data and wrote the report. CD and LL: checked the data and results. SY: revised the report from preliminary draft to submission. All authors have read and approved the manuscript.

## Conflict of Interest

The authors declare that the research was conducted in the absence of any commercial or financial relationships that could be construed as a potential conflict of interest.
