# Supplementary material for: Epidemiological Trends and Hotspots of Other Infectious Diarrhea (OID) in Mainland China: A Population-Based Surveillance Study From 2004 to 2017
Source: Front Public Health. 2021 Jul 22;9:679853. doi: 10.3389/fpubh.2021.679853 (PMC8339203; doi:10.3389/fpubh.2021.679853)
Supplement: Supplementary file 1 [file Table_1.DOC]

**Supplemental Table 1**. Incidence and trends of other infectious diarrhea by 31 surveillance provinces or areas in China, 2004–2017

| **Areas** | **Number of cases** | **Average annual incidence (per 100 000 people)** | **Number of deaths** | **Case-fatality ratios** **(per 100 000 people)** | **Annual percentage change (%) (95% CI)** | | | | **Trend** |
| --- | --- | --- | --- | --- | --- | --- | --- | --- | --- |
| Tianjin | 531725 | 289.33 | 0 | 0 | 2004-2006:116.50 (28.10-265.92)* | 2006-2010:-16.88(-29.45--2.09)* | | 2010-2017:-1.83(-6.51-3.10) | Decrease |
| Beijing | 672875 | 253.67 | 24 | 3.57 | 2004-2006:15.88 (-1.99-37.02) | 2006-2009:-13.98(-26.65-0.89) | | 2009-2017:-4.50(-6.32--2.65)* | Decrease |
| Zhejiang | 1485164 | 200.34 | 21 | 1.41 | 2004-2017:-0.24 (-2.62-20.19) | | | | Stable |
| Ningxia | 109170 | 122.69 | 1 | 0.92 | 2004-2007:16.62 (-2.50-39.48) | | 2007-2017:1.17 (-1.08-3.46) | | Increase |
| Guangdong | 1554709 | 109.99 | 69 | 4.44 | 2004-2017:4.03 (1.30-6.84)* | | | | Increase |
| Anhui | 776078 | 90.57 | 12 | 1.55 | 2004-2017:13.48 (11.38-15.02)* | | | | Increase |
| Chongqing | 345359 | 84.05 | 20 | 5.79 | 2004-2017:9.23 (7.26-11.24)* | | | | Increase |
| Guangxi | 540031 | 81.05 | 107 | 19.81 | 2004-2014:3.61 (-0.32-7.70) | | 2014-2017:46.19 (24.74-71.33)* | | Increase |
| Xinjiang | 217932 | 70.77 | 25 | 11.47 | 2004-2006:43.68 (4.29-97.93)* | | 2006-2017:-3.41 (-5.00--1.80)* | | Decrease |
| Hebei | 695342 | 69.28 | 20 | 2.88 | 2004-2006:54.00 (15.94-104.57)* | 2006-2009:-4.88(-14.13-5.58) | | 2009-2017:2.44(-0.29-5.26) | Increase |
| Jiangxi | 366055 | 58.64 | 44 | 12.02 | 2004-2007:24.00 (0.30-53.31) | | 2007-2017:4.09 (1.69-6.54)* | | Increase |
| Fujian | 288764 | 55.77 | 21 | 7.27 | 2004-2017:7.54 (4.66-10.50)* | | | | Increase |
| Shaanxi | 286584 | 54.43 | 23 | 8.03 | 2004-2017:6.78(4.54-9.07)* | | | | Increase |
| Qinghai | 37779 | 47.6 | 5 | 13.23 | 2004-2017:11.50(7.35-15.81)* | | | | Increase |
| Shandong | 593378 | 44.21 | 7 | 1.18 | 2004-2017:13.46 (11.16-15.82)* | | | | Increase |
| Sichuan | 492856 | 42.95 | 38 | 7.71 | 2004-2006:18.41 (-8.33-52.93)* | 2006-2015:-6.56(-9.31--3.73)* | | 2015-2017:14.95(-13.39-52.56) | Decrease |
| Hubei | 337350 | 41.62 | 8 | 2.37 | 2004-2006:217.39 (-20.65-1169.49) | | 2006-2017:12.53 (10.22-14.88)* | | Increase |
| Henan | 547022 | 41.32 | 23 | 4.2 | 2004-2017:13.37 (9.19-13.58)* | | | | Increase |
| Hainan | 47332 | 38.71 | 1 | 2.11 | 2004-2017:4.47 (1.16-7.90)* | | | | Increase |
| Shanxi | 186099 | 37.67 | 11 | 5.91 | 2004-2017:6.63 (4.09-9.22)* | | | | Increase |
| Gansu | 127861 | 35.12 | 9 | 7.04 | 2004-2017:11.01 (8.86-13.21)* | | | | Increase |
| Liaoning | 210426 | 34.67 | 2 | 0.95 | 2004-2017:7.47 (3.37-11.73)* | | | | Increase |
| Hunan | 302910 | 32.85 | 24 | 7.92 | 2004-2009:22.11 (14.29-30.46)* | | 2009-2017:3.48 (1.25-5.77)* | | Increase |
| Yunnan | 162354 | 25.14 | 13 | 8.01 | 2004-2017:15.05 (12.19-18.00)* | | | | Increase |
| Shanghai | 71123 | 23.61 | 0 | 0 | 2004-2008:6.39 (-9.21-24.66)* | 2008-2011:-17.03(-49.20-35.52) | | 2011-2017:9.24(1.12-18.00)* | Stable |
| Guizhou | 103023 | 20.26 | 30 | 29.12 | 2004-2017:7.52 (5.54-9.53)* | | | | Increase |
| Heilongjiang | 197218 | 18.08 | 3 | 1.52 | 2004-2017:1.54 (-1.47-4.64) | | | | Stable |
| Jilin | 69811 | 13.05 | 7 | 10.03 | 2004-2012:28.91 (22.97-35.14)* | 2012-2015:-32.65(-52.33--4.85) | | 2015-2017:7.05(-29.95-63.60) | Stable |
| Jiangsu | 36611 | 10.64 | 5 | 13.66 | 2004-2009:-7.86 (-18.46-4.11) | | 2004-2009:9.74 (4.21-15.56)* | | Increase |
| Inner Mongolia | 19806 | 5.17 | 1 | 5.05 | 2004-2006:167.64 (-30.46-930.11) | 2006-2009:-9.86(-47.21-53.94) | | 2009-2017:15.41(10.42-20.62)* | Increase |
| Tibet | 1500 | 3.55 | 0 | 0 | 2004-2017:-13.11 (-20.33--5.24) | | | | Decrease |

**Supplemental Table 2. Global autocorrelation analysis of national** **other infectious diarrhea, 2004–2017**

| **Year** | **Moran’s I** | **Z** | **P** | **Correlation** |
| --- | --- | --- | --- | --- |
| **2004** | 0.2510 | 2.8634 | 0.01 | Positive correlation |
| **2005** | 0.3957 | 3.9983 | 0.004 | Positive correlation |
| **2006** | 0.4106 | 4.3987 | 0.004 | Positive correlation |
| **2007** | 0.6183 | 5.3692 | 0.01 | Positive correlation |
| **2008** | 0.3681 | 3.6157 | 0.006 | Positive correlation |
| **2009** | 0.4297 | 4.2277 | 0.004 | Positive correlation |
| **2010** | 0.3847 | 3.5946 | 0.005 | Positive correlation |
| **2011** | 0.3327 | 3.0732 | 0.006 | Positive correlation |
| **2012** | 0.3384 | 3.1751 | 0.006 | Positive correlation |
| **2013** | 0.3185 | 2.9931 | 0.007 | Positive correlation |
| **2014** | 0.3387 | 3.1533 | 0.007 | Positive correlation |
| **2015** | 0.2973 | 2.7462 | 0.009 | Positive correlation |
| **2016** | 0.2016 | 1.9258 | 0.042 | Positive correlation |
| **2017** | 0.2081 | 1.9902 | 0.0350 | Positive correlation |
